# Mechanically Controlled Electron Transfer in a Single-Polypeptide Transistor

**DOI:** 10.1038/srep39792

**Published:** 2017-01-04

**Authors:** Sheh-Yi Sheu, Dah-Yen Yang

**Affiliations:** 1 Department of Life Sciences, Institute of Genome Sciences and Institute of Biomedical Informatics, National Yang-Ming University, Taipei 112, Taiwan; 2 Institute of Atomic and Molecular Sciences, Academia Sinica, Taipei 106, Taiwan

## Abstract

Proteins are of interest in nano-bio electronic devices due to their versatile structures, exquisite functionality and specificity. However, quantum transport measurements produce conflicting results due to technical limitations whereby it is difficult to precisely determine molecular orientation, the nature of the moieties, the presence of the surroundings and the temperature; in such circumstances a better understanding of the protein electron transfer (ET) pathway and the mechanism remains a considerable challenge. Here, we report an approach to mechanically drive polypeptide flip-flop motion to achieve a logic gate with ON and OFF states during protein ET. We have calculated the transmission spectra of the peptide-based molecular junctions and observed the hallmarks of electrical current and conductance. The results indicate that peptide ET follows an NC asymmetric process and depends on the amino acid chirality and α-helical handedness. Electron transmission decreases as the number of water molecules increases, and the ET efficiency and its pathway depend on the type of water-bridged H-bonds. Our results provide a rational mechanism for peptide ET and new perspectives on polypeptides as potential candidates in logic nano devices.

Protein electron transfer (ET) plays a crucial role in diverse biological systems, including signal transduction, respiration and photosynthesis^1^,^2^,^3^. Because of their structural and functional versatility^4^,^5^,^6^,^7^, proteins are particularly amenable as molecular building blocks of functional nano-devices for biosensors, quantum computers and bioelectronics^8^,^9^,^10^,^11^,^12^,^13^,^14^,^15^,^16^. Understanding the processes involved in protein ET is important not only to unravel key biological functions but such findings will also help to apply proteins when designing nanoscale molecular electronics. Many extensive studies of protein ET have been performed experimentally^17^,^18^,^19^,^20^,^21^,^22^ and theoretically^23^,^24^. The ET between the redox centers mediated by peptide bridges has been thought to involve two possible mechanisms: the superexchange model and electron hopping model^25^,^26^,^27^,^28^,^29^. In the superexchange (or tunneling) mechanism, ET takes place via coupling between the virtual states of the bridging units and involves tunneling movement through the bridge part without a transient stay in the bridge state; in such circumstances the rate constant shows an exponential decaying function of the peptide length^30^. This mechanism can thus be described as having the decay parameter β that is dependent on the bridge length, the conformational rigidity and the electronic properties of the electron donor and acceptor^31^,^32^,^33^,^34^. Once a peptide exceeds a certain length, the ET process has been interpreted as undergoing a crossover from the tunneling mechanism to a hopping mechanism^27^,^35^,^36^. The electron-hopping mechanism of ET involves oxidized or reduced intermediates that act via a multistep process wherein the electron (or charge) hops using intermediate sites as stepping stones^34^. In addition, several experimental studies have been devoted to investigating the mechanism of ET caused by the structural fluctuations of the molecular bridge^37^,^38^,^39^,^40^. Nevertheless, the exact mechanism of peptide ET has remained in debate.

In order to unravel the quantum transport, rather than studying ET rate^26^,^41^, the electrical conductance and I-V curve of molecules have been measured using molecular junction techniques^13^,^15^,^16^,^42^,^43^. Atomic force microscopy and scanning tunneling microscopy as techniques have greatly contributed to the investigation of protein electron transmission^20^,^44^,^45^. Peptide ET seems to be mainly controlled by the sequence of the peptide and its secondary structure rather than chain length^14^,^16^,^27^,^29^,^46^. The regular H-bonds between the main-chain N and O atoms within the secondary structures of peptides are expected to function as ET pathways^27^,^29^,^47^,^48^,^49^. Furthermore, ET within a helical peptide is direction dependent^50^. Proline contains a unique cyclic side chain linked to the backbone and thus this amino acid is more structurally rigid than other amino acids and as a result is unable to form intramolecular H-bonds. Thus, although proline cannot function as a relay station due to the fact that it is difficult to oxidize its side chain, it is still able to promote the ET process^51^, and helical polyproline bridges exhibit a high ET rate. Finally, the ET process strongly relies on protein dynamics, which is inevitably affected by the water^52^,^53^,^54^,^55^,^56^. Protein ET in water has been found to be distance dependent and has a low efficiency with a decay constant β-value that is close to 1.0 and 1.3 Å^−1^ for α-helices and β-sheets, respectively^57^; thus water is not a good solvent for protein ET.

Quantum transport measurements produce conflicting results because of the technical limitations associated with them; these make it difficult to precisely determine molecular orientation, the nature of the moieties, the presence of the surroundings and the temperature. These experiments have mainly focused on peptide bundles and they lack an atomic scale quantum mechanics interpretation; thus an understanding of the protein ET pathway and the mechanism involved remains a considerable challenge. As a result of the above, the successful use of proteins in nano devices will require more advanced explorations of the detailed mechanism at an atomic level in order to determine the efficiency of electron transmission and the correlation between electronic properties and specific structural features.

In the present study, using a setup consisting of a single molecular junction, protein electrical conductivity was determined via a piece-by-piece calculation. The same mechanism can then simply be repeated for longer peptide chains. Various tripeptides were studied systematically using a combination of density functional theory^24^ and non-equilibrium Green’s function formalism (DFT-NEGF) to calculate the molecule’s electron transmission spectrum (TS). The TS intensity close to the Fermi energy level (E_F_) resolves the electrical conductance depending on the band gap Δ and the density of state (DOS). Here, we have directly demonstrated that peptide ET is largely dependent on the intrinsic structures of the peptide. Our results confirm that the ET pathway in peptides occurs through-bonds rather than through-space. A unique through-bond ET occurs when the distance *d*_*O*–*O*_ between adjacent carbonyl groups on the peptide backbone is less than a critical *d*_*c*_ value of 2.03** **Å. Notably, the electron does not pass through the regular H-bonds in well-defined secondary structures; however, an absorbed anion close to these H-bonds would seem to facilitate ET. The H-bond networks between water molecules and the peptide can tremendously alter ET efficiency.

## Results and Discussion

### NC asymmetry ET and distance-dependent conductance

**Figure 1:**
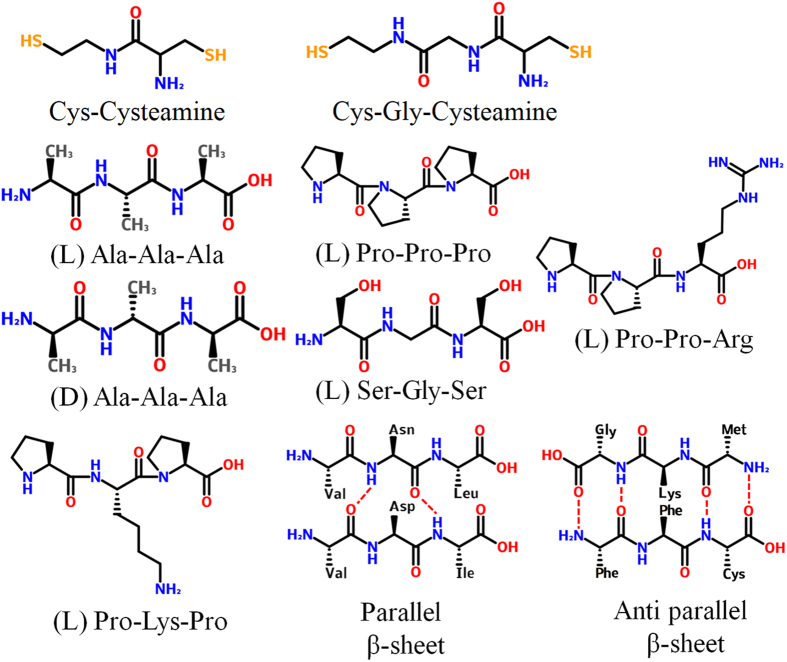
Chemical structures of the peptides.

**Figure 2:**
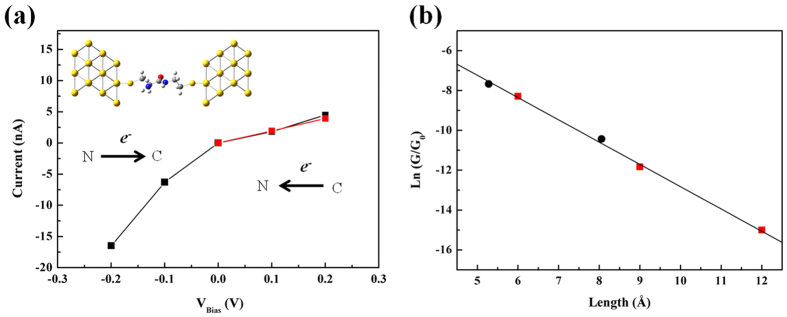
I-V curve and conductance of the peptides. (**a**) I-V curve of the peptide [Cys-Cysteamine]. A single molecular junction: peptide (O: red, N: blue, C: gray and H: light gray) was wired to the Au electrodes (yellow) through the interfacial S atom (brown). The source, scatter and drain components are the left electrode, the peptide and the right electrode, respectively. The curve of ^N^[Cys-Cysteamine]^C^ (N ← C type, black square) and the experimental data^13^ (red square) are shown. In the positive (negative) V_bias_ region, the electron flow direction is denoted as 
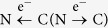
. (**b**) The conductance G of the peptides [Cys-Cysteamine] and [Cys-Gly-Cysteamine]: our result (black circle) and the experimental data^13^ (red square). The structure was optimized at *d*_*O*–*O*_ = 5.0 Å because the peptide was stretched in the experiments.

The I-V curve of the dipeptide [Cys-Cysteamine] was calculated and compared with experimental results^13^,^58^ (Figs 1 and 2a). The magnitude of the electronic flow of the N → C direction at the bias voltage V_bias_ = −0.1 V was 3.5 times greater than that of the reversed N ← C direction at V_bias_ = 0.1 V, where the voltage is within the ohmic regime. This flow is thus referred to as an NC asymmetry ET process. Compared with our computation, the experimental data are in the same range as that in the N ← C direction. This result illustrates that the peptide ET process is direction-dependent, consistent with electrical measurements^50^. The length dependence of conductance G for the two peptides [Cys-Cysteamine] and [Cys-Gly-Cysteamine] was also calculated (Fig. 2b), and then was fitted by G = Aexp(−βr), where A is a pre-factor, β is a distance-dependent constant and r is the peptide length. Our calculated β-value of 1.0 Å^−1^ is also in agreement with experimental results^13^. Here, the NC asymmetry is by reason that the electronic energy jump between the neighboring amino acids was from 0.07 to 0.50 eV experimentally^59^, and even a pair of two identical amino acids had about 0.6 eV difference due to the natural asymmetry of the C-side and the N-side of each amino acid^60^.

### Effects of L/D enantiomers and α-helical handedness

**Figure 3:**
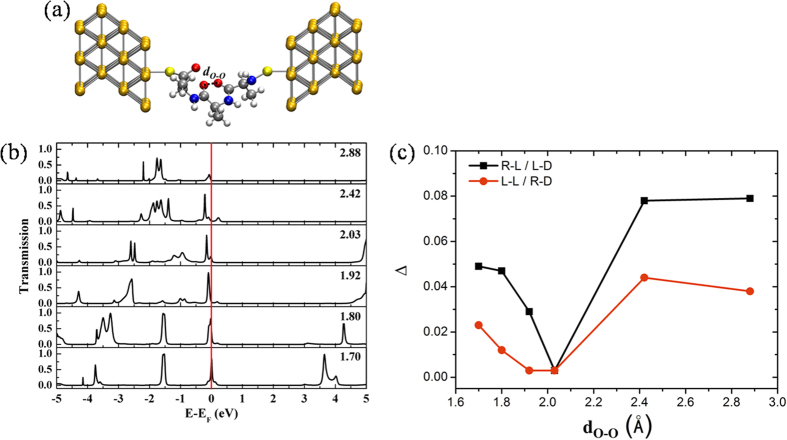
Transmission analysis of R-L(Ala)_3_. (**a**) A single molecular junction. The notations are identical to those in the legend of Fig. 2. (**b**) TS at *d*_*O*–*O*_: 1.70, 1.80, 1.92, 2.03, 2.42 and 2.88 Å. (**c**) Energy gap Δ versus *d*_*O*–*O*_.

To determine whether ET depends on the optical isomer of the peptides, four enantiomers of a tripeptide (Ala)_3_ were examined. In the notation X-Y(Ala)_3_, X denotes a left-handed (L) or right-handed (R) α-helical structure, and Y is the L or D enantiomer of an amino acid (Fig. 1). Each configuration was at the designated distance *d*_*O*–*O*_ between the two O atoms of the adjacent carbonyl groups, and TS was calculated (Figure S1a–d). The TS results were identical for mirror images, i.e., (L-L(Ala)_3_, R-D(Ala)_3_) and (L-D(Ala)_3_, R-L(Ala)_3_). However, the TS(D_e_) intensities of (L-D(Ala)_3_, R-L(Ala)_3_) were higher than those of (L-L(Ala)_3_, R-D(Ala)_3_) within the energy range D_e_. This difference demonstrates that the electron transmission of peptides depends on the amino acid chirality and α-helical handedness. The TS of R-L(Ala)_3_ with respect to *d*_*O*–*O*_ is shown in Fig. 3a,b; the TS(D_e_) peak is sharp at *d*_*O*–*O*_ < 2.03 Å but shifts away from D_e_ at *d*_*O*–*O*_ > 2.03 Å. As shown in Fig. 3c, there is a minimum Δ value at *d*_*c*_ = 2.03 Å, indicating that *d*_*c*_ is a critical distance permitting ET. The conductance abruptly decreases with increasing *d*_*O*–*O*_ while stretching the peptide length. Note that *d*_*O*–*O*_ is modulated by protein dynamics and redox potential^61^. Remarkably, the TS(D_e_) intensity decreases as *d*_*O*–*O*_ increases and strongly depends on *d*_*O*–*O*_, creating a shorter pathway for ET in peptides.

The molecular orbital structures of R-L(Ala)_3_ at *d*_*O*–*O*_ = 1.92 Å reveal an apparent migration of the electron density distribution from the HOMO to the extended LUMO across the molecular junction and the interface S atom of the drain component (Figure S1e). Notably, although the V_bias_ is equal to zero, charge separation remains in the system. The coefficients of the atomic orbitals for the HOMO and LUMO of R-L(Ala)_3_ at two *d*_*O*–*O*_ values are listed (Table S1a and S1b). At *d*_*O*–*O*_ = 1.92** **Å, all atomic orbital coefficients of the eigenchannel 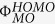 are smaller than that of 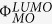, implying that the electron density migrates from the HOMO to the LUMO; the corresponding atomic orbital structures are shown (Figure S1f). By contrast, at *d*_*O*–*O*_ = 2.42** **Å, there is no electron density change between the HOMO and LUMO (Figure S1g). This result illustrates that ET occurs through the 2p-π orbital overlap between the two O atoms of the nearby carbonyl groups.

### H-bonds in the secondary structure of peptides

Next, we calculated the TS of three residues (Ala^405^
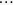
Arg^409^
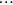
His^413^) extracted from an α-helical protein (PDB: 4nl4)^62^ with a rigid intramolecular H-bond pitch 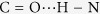. As is evident in Figure S2a, there is no TS(D_e_) peak for this structure. Similar results have been obtained for β**-**sheets, both parallel (β_1_: ^N^Val-Asp-Ile^C^ and β_2_: ^N^Val-Asn-Leu^C^) and anti-parallel (β_1_: ^N^Met-Lys-Gly^C^ and β_2_: ^C^Cys-Phe-Phe^N^), extracted from a protein structure (PDB: 1nwo)^63^ with two and four intermolecular H-bonds, respectively (Fig. 1, Figures S2f and S2i). Molecular orbital analyses revealed that the electron does not transfer from one electrode to the other one, i.e., there is no charge separation (Figure S2l). This implies that at *d*_*O*–*O*_ ≫ *d*_*c*_ or without the 2p-π orbital overlap between the two O atoms, these rigid secondary structures could not conduct electron. Experimental result showed that even the electron transfers via a nearby paired H-bonds^64^. However, for example in the β-sheet the electron passes through the first paired H-bonds from the polypeptide chain β_2_ to the chain β_1_, but it does not transfer further along the chain β_1_ because the carbonyl groups are constrained by the H-bond, leading to *d*_*O*–*O*_ ≫ *d*_*c*_. Hence, the mechanism of ET excludes the process through the H-bond in the rigid secondary structure of peptides in the gas phase, conflicting with the experimental results^65^,^66^,^67^,^68^. The extent to which peptide ET occurs in experiments is not straightforward. Below, we demonstrate that it is possible to resolve this discrepancy.

Many metal protein-modifying agents, such as Chloropentaamineruthenium (III) dichloride RuCl(NH_3_)_5_Cl_2_^69^, are widely used as redox reagents^70^. However, the role of a counter anion, for example the Cl^−1^ anion, in mediating ET is unclear for this reagent. We therefore adopted a more realistic system by adding Cl^−1^ ions and water molecules near the carbonyl group of these peptides and performed the TS calculation. Interestingly, a TS(D_e_) peak was observed for the systems in the presence of the Cl^−1^ ions, indicating that the 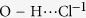 H-bond contributes significantly to ET (Figure S2b,c,d,g and j). The TS intensity increases extraordinarily as the number of Cl^−1^ ions increases. However, no TS(D_e_) peak was observed for the systems with only added water molecules (Figure S2e,h and k). Thus, in electrolyte solutions, even for the well-defined secondary structures of peptides, the adsorbed counter anion plays a crucial role in ET.

### Proline pair effect

**Figure 4:**
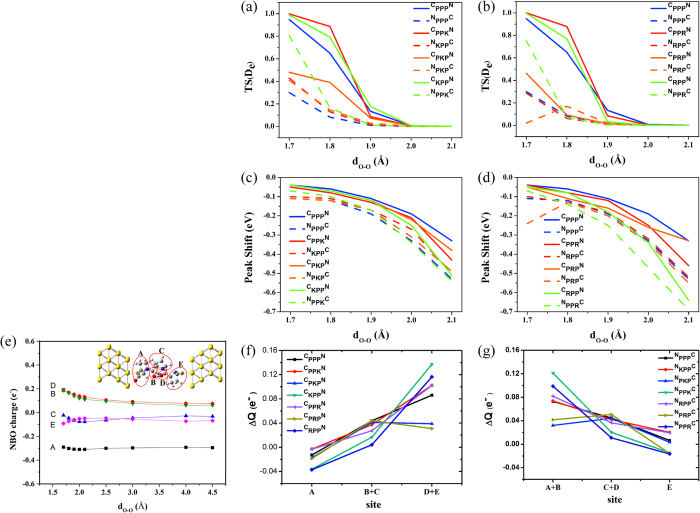
Transmission analysis of proline-based peptides. (**a**) and (**b**) TS(D_e_) intensity versus *d*_*O*–*O*_ for the peptides PPP, PKP, KPP, PPK, PRP, RPP and PPR. (**c**) and (**d**) Shift of the TS(D_e_) peak versus *d*_*O*–*O*_. **(e)** NBO charge of the ^C^PPP^N^ versus *d*_*O*–*O*_ in the five regions of A, B, C, D and E. The notations of the molecular junction structure are the same as those in the legend of Fig. 2. The ΔQ versus the site of the peptide with (**f**) C → N type and (**g**) N ← C type.

Because of the unique cyclic backbone structure of proline, the electronic properties of proline-rich peptides are of interest to determine whether this structure permits electron delocalization in ET. We studied the proline-based tripeptides PPP, XPP, PXP, and PPX, where X is a polar residue with a long side chain, i.e., Lys (K) or Arg (R), to calculate the electron transmission (Fig. 1 and Figure S3). For these peptides, TS(D_e_) intensity decreases and the peak position shifts far from the E_F_ with increasing *d*_*O*–*O*_ (Fig. 4a–d). The C ← N type has a higher TS(D_e_) intensity and a smaller peak shift compared with the N ← C type. Once the PP pair is broken, such as PXP, its TS(D_e_) greatly decreases. Hence, the PP pair is superior to the other pairs for conducting electrons.

Gaussian natural bond orbital (NBO) analysis^71^ was also performed to calculate the natural charge (Q) of each group versus *d*_*O*–*O*_ (Fig. 4e and Figure S3b). The Q change is defined as 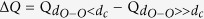, where the positive (negative) ΔQ value reflects the loss (gain) of electronic charge for each group at *d*_*O*–*O*_ < *d*_*c*_. If ΔQ ≈ 0, then no electron resides at this site. In the C ← N type, ΔQ of these peptides except for PXP follows the trend ΔQ^*A*^ < ΔQ^*B*+*C*^ < ΔQ^*D*+*E*^, where ΔQ^*S*^ denotes ΔQ at the S site (Fig. 4f). A decreasing ΔQ corresponds to electron transport from the N terminus to the C terminus as *d*_*O*–*O*_ decreases from 4.50 to 1.90 Å; notably, this flow direction is consistent with that from the drain component to the source component. Both ΔQ^*B*+*C*^ and ΔQ^*D*+*E*^ of the above proline peptides are positive because the electron resides at the A site (C terminus) before entering the source component. By contrast, the ΔQ^*A*^ values are negative and follow the order ^C^XPP^N^ (−0.036 *e*^−^) < (^C^PPP^N^ and ^C^PXP^N^) (−0.016*e*^−^) < ^C^PPX^N^ (−0.003*e*^−^), implying that X at the C terminal side is more likely than proline to attract an electron. Considering the PP pairing effect in ^C^PXP^N^, ΔQ follows the order ΔQ^*A*^ < ΔQ^*D*+*E*^ < ΔQ^*B*+*C*^ due to the electrochemical reduction of the middle X, which restricts the ET efficiency. More concrete evidence of this effect is that a larger difference ΔΔQ = ΔQ^*D*+*E*^ − ΔQ^*A*^ reflects a larger charge separation and a higher efficiency of electron transmission. In addition, the ΔQ values of peptides of the N ← C type decrease from the N terminus to the C terminus (Fig. 4g), identical to the electron flow direction. Because the electron flow is from the drain component to the source component, the reduced intensity of the electron transmission of the N ← C type is due to the electronic charge offset of the reversed ΔQ distribution. The ΔQ value of the N ← C type is smaller than that of the C ← N type. These results confirm that the electron preferentially resides at the C terminus, i.e., it displays NC asymmetry.

Here, we have demonstrated that the PP pair can facilitate ET. The direction of ET is from the N-terminus to the C-terminus, regardless of how the N-terminus of the peptide is connected to the source component or the drain component. Although the ionization potential (IP) of the amino acids follows the order Arg ~ Lys > Pro^72^, our studies indicate that the IP does not affect the peptide ET process and that the TS depends on the X position rather than the X type.

### Solvation effect

**Figure 5:**
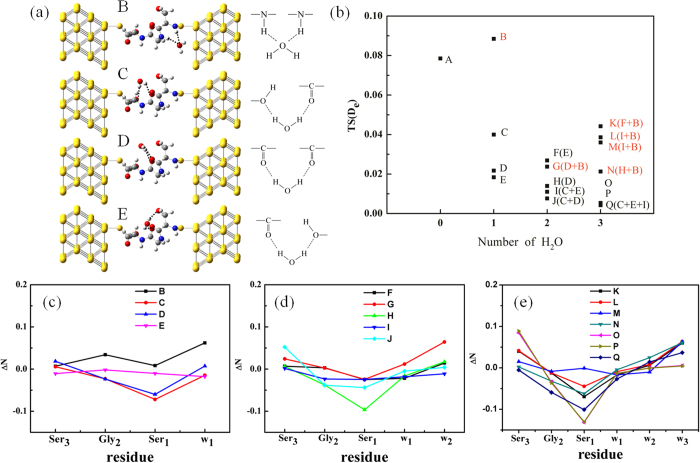
Transmission analysis of the peptide SGS with water molecules. (**a**) Plots of molecular junctions and water-bridged H-bond types, B (NHO), C (OHO), D (COHO) and E(OHO). The notations are identical to those in the legend of Fig. 2. A water molecule is shown in a ball-and-stick representation and color-coded by atom type. (**b**) TS(D_e_) intensity versus the number of H_2_O molecules. The system contains NHO-type water-bridges (colored in red), and the water-bridge types are indicated in parentheses. The number of water molecules dependence of ΔN is (**c**) SGS + H_2_O, (**d**) SGS + 2 H_2_O and **(e)** SGS + 3 H_2_O.

To study the solvation effect on peptide ET, we considered a system composed of a polar tripeptide Ser-Gly-Ser (SGS) and a few water molecules, which were added individually until reaching three molecules. The SGS molecular junction was established in a C ← N direction, and the torsion angles of the Ser_1_ and Gly_2_ were designated as *d*_*O*–*O*_ = 1.96 Å. The detailed structures and their TS values are shown in Figure S4. **TS(D**_**e**_) versus the number of water molecules and the natural charge difference, ΔN = (the natural charge of SGS + n H_2_O, n = 1, 2 and 3) − (the natural charge of SGS), between the peptide and its reference system A, which consists of the peptide alone, are shown in Fig. 5. More generally, the water molecule is near the carbonyl group and the amide group of the peptide, resulting in the formation of three typical water-bridged H-bonds: -NH…O_W_…HN- (NHO type), -C = O…H-O_W_-H…O = C- (COHO type) and -C = O…H-O_W_-H…O-H (OHO type). Here, O_W_ is the O atom of the water molecule, as shown in structures B, C, D and E in Fig. 5a. We examined the effect of these H-bonds on TS(D_e_) and found that TS(D_e_) decreases as the number of water molecules increases (Fig. 5b). The TS(D_e_) of system B is higher than that of system A because system B contains one more NHO-type water-bridge than system A. However, TS(D_e_) decreases in the COHO-type (system D) and the OHO-type (systems C and E) H-bonds. With more than one water molecule, the water-bridge is a combination of these three H-bond types; for example, system F contains types COHO and OHO, and system G contains types NHO and COHO. Importantly, if the systems, e.g., G, K, L, M and N, contain the NHO-type, then their TS(D_e_) values are slightly higher than those of the systems containing only types COHO or OHO with the same number of water molecules. The I, J, O, P and Q systems contain only pure COHO and OHO types, and their TS(D_e_) values are greatly reduced. Thus, TS(D_e_) is dependent on the type of water-bridge in peptide ET.

To provide additional insight into the transport properties of the different configurations of the water-bridged H-bond network, we analyzed the electronic charge distribution in terms of ΔN. The ΔN values of the three residues for system B are positive (Fig. 5c), revealing an enhancement of electron transport through the NHO-type network. However, the TS(D_e_) reduction correlates well with the ΔN variation in the C, D and E systems (Fig. 5b,c); their ΔN values are negative and decrease from the C-terminus (Ser_3_) to the N-terminus (Ser_1_), and thus the electron prefers to remain at the N-terminus and has a reduced TS(D_e_). In particular, Ser_3_ in system E has a negative ΔN; thereby, the electron is trapped at this site and is not transferred to the source component. NBO analysis of these systems with more water molecules revealed similar results (Fig. 5d,e). In particular, the COHO and OHO types can reverse the direction of the electrical current and depress electron transmission. The NBO charge of the water molecule in these configurations follows the order NHO ≫ COHO > OHO (Table S2). Notably, the natural charge value of the NHO (OHO) type is positive (negative), indicating that the water molecule loses (gains) an electron much easier compared with the peptide alone; however, the COHO type has a smaller positive value. This smaller value is attributable to the 2p-π orbital overlap as well as the electronegativity difference. The NHO type water-bridge provides a constructive and additional pathway for ET. But, the COHO type eventually has less electrical conductivity than the NHO type. Hence, we confirmed that water molecules can not only facilitate but also depress ET in peptides, being conditional ET. These observations are consistent with experimental results^73^. The low ET efficiency of peptides in aqueous solution is mainly due to unfavorable ET pathways.

### Logic gate

**Figure 6:**
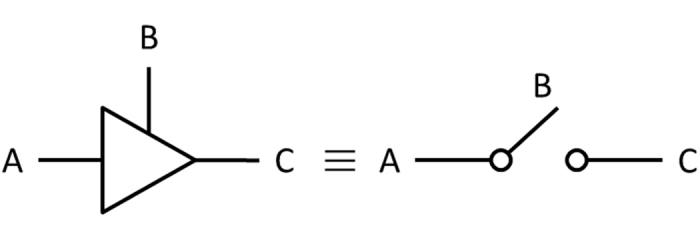
Schematic three-state logic gate. The tri-state buffer (left) is equivalent to a switch (right). There are ON and OFF states for the three-state logic gate. A and C are the junctions, and B is the gate.

A unique through-bond ET occurs at *d*_*O*–*O*_ < *d*_*c*_, causing a residue to be in the logic ON state; otherwise, it is in the logic OFF state. Accordingly, it is clear that each residue can act as a three-state logic gate (Fig. 6). There is a typical rotation rate for the amino acid torsion angles (φ, ψ) on the time scale of 100 fs^74^. The plausible triggering mechanism is the electron injected from the electrode part into the polypeptide residues to provide energy to the rotational degrees of freedom, and thus allow the *d*_*O*–*O*_ < *d*_*c*_. Hence, a polypeptide can behave as a series logic gate element, and its electrical conductivity can be switched in the sub-picosecond regime.

## Conclusion

In summary, molecular electronics have undergone rapid development, and their potential applications continuously surprise scientists and engineers. Functional polypeptide molecules are attractive for designing bioelectronics. Many distinct electronic transport behaviors are understood, but the elucidation of the novel intrinsic mechanism and the functionality of individual molecules remains a formidable challenge and cannot be ignored. This work provides a two-way approach of using molecular junction techniques to unravel biomolecular ET mechanism and using proteins with striking functionality in molecular electronics and spintronics^75^. The results of the present study suggest that the electronic properties of a peptide-based molecular junction can be mechanically controlled by protein conformation, motion and environment. Furthermore, protein functionality provides a well-crafted design for the construction of ultrafast sub-picosecond-scale molecular switches and integrated circuits due to the protein H-bond network. The use of peptide-based logic elements will be advantageous for the design of new molecular electronic devices.

### Computational method

All DFT calculations were performed using the Gaussian 09 program^71^. The system was constructed with a peptide chain wired to two Au-electrodes through the tip’s S atoms. The electrode, i.e., source and drain, consisted of four layers of a Au 3 × 3 lattice along the Au(111) direction, and the tip’s S atom was 2.32 Å from the Au interface. The geometries of all systems were optimized at the B3LYP level. The 6–31 G(d, p) basis set was used for the C, H, N and O atoms, whereas the LANL2DZ basis set was used for Au atoms. We performed *ab initio* quantum calculations to optimize the geometry at various *d*_*O*–*O*_ values between the two O atoms of the carbonyl groups of adjacent amino acids. An ALACANT program^76^, which follows an onion shell structure to construct the device part and the far bulk electrode parts, was used to calculate electron TS based on the DFT-NEGF theory. The electronic structures of the polypeptides and the Au electrodes (the device part) were computed at the DFT local density approximation level with a minimal basis set. A semi-empirical tight-binding Bethe lattice model was used for the far bulk electrode parts. The convention used is that the C ← N (N ← C) type of molecular junction denotes the N-terminus (C-terminus) of the peptide wired to the right electrode, i.e., source -^C^peptide^N^- drain (source -^N^peptide^C^- drain).

According to the Landauer formula^77^,^78^, the conductance G can be obtained as follows:


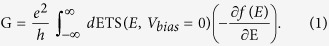


The I-V curve can be obtained as follows:


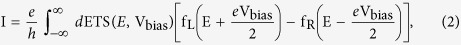


where *h* is Planck’s constant, *e* is the electrical charge, E is the energy, f(E) is the Fermi-Dirac distribution, TS(E, V_bias_) is the transmission spectrum, I is the electrical current and V_bias_ is the bias voltage. Because the Fermi-Dirac distribution is close to a step function and its derivative is a sharp bell-like function centered at E_F_, the contribution of TS(E,V_bias_) to the electrical current and conductance depends on the TS(E_F_) value at V_bias_ = 0. The Δ value is the band gap between the lowest unoccupied molecular orbital (LUMO) and the highest occupied molecular orbital (HOMO). When the Δ value is less than 0.05 eV and when the DOS around the E_F_ covers both the HOMO and LUMO energy levels, a TS peak appears within the range of 
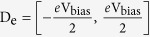
. Under this circumstance, the delocalized electron density distribution in the molecular junction leads to a tendency for electron tunneling of these systems, i.e., a TS(D_e_) peak exists. Importantly, protein ET is driven by the redox potential, as in membrane proteins^68^ and metalloproteins^65^. The TS(D_e_) peak is typically generated by electron tunneling between the HOMO and LUMO and is irrelevant to the other intrinsic molecular energy levels^79^,^80^,^81^; therefore, the peak indicates plausible electrical conductivity or current. Otherwise, if TS(D_e_) is zero, then the electron cannot tunnel through the peptide.

## Additional Information

**How to cite this article**: Sheu, S.-Y. and Yang, D.-Y. Mechanically Controlled Electron Transfer in a Single-Polypeptide Transistor. *Sci. Rep.* **7**, 39792; doi: 10.1038/srep39792 (2017).

**Publisher's note:** Springer Nature remains neutral with regard to jurisdictional claims in published maps and institutional affiliations.

## Supplementary Material

Supplementary Data

## References

[b1] Bashir Q., Scanu S. & Ubbink M. Dynamics in electron transfer protein complexes. FEBS J. 278, 1391–1400 (2011). 21352493 10.1111/j.1742-4658.2011.08062.x

[b2] Kulawiak B. . The mitochondrial protein import machinery has multiple connections to the respiratory chain. BBA. Bioenergetics 1827, 612–626 (2013). 23274250 10.1016/j.bbabio.2012.12.004

[b3] Kamran M. . Demonstration of asymmetric electron conduction in pseudosymmetrical photosynthetic reaction centre proteins in an electrical circuit. Nat. Commun. 6, 6530 (2015). 25751412 10.1038/ncomms7530 PMC4366537

[b4] Cavalli S., Albericio F. & Kros A. Amphiphilic peptides and their cross-disciplinary role as building blocks for nanoscience. Chem. Soc. Rev. 39, 241–263 (2010). 20023851 10.1039/b906701a

[b5] Chen C.-L. & Rosi N. L. Peptide-based methods for the preparation of nanostructured inorganic materials. Angew. Chem. Int. Edit 49, 1924–1942 (2010). 10.1002/anie.200903572 20183835

[b6] Hauser C. A. E. & Zhang S. Designer self-assembling peptide nanofiber biological materials. Chem. Soc. Rev. 39, 2780–2790 (2010). 20520907 10.1039/b921448h

[b7] Lakshmanan A., Zhang S. & Hauser C. A. E. Short self-assembling peptides as building blocks for modern nanodevices. Trends Biotechnol. 30, 155–165 (2012). 22197260 10.1016/j.tibtech.2011.11.001

[b8] Aradhya S. V. & Venkataraman L. Single-molecule junctions beyond electronic transport. Nat. Nano. 8, 399–410 (2013). 10.1038/nnano.2013.91 23736215

[b9] Choi Y. . Single-molecule dynamics of lysozyme processing distinguishes linear and cross-linked peptidoglycan substrates. J. Am. Chem. Soc. 134, 2032–2035 (2012). 22239748 10.1021/ja211540z PMC3271187

[b10] Choi Y. . Single-molecule lysozyme dynamics monitored by an electronic circuit. Science 335, 319–324 (2012). 22267809 10.1126/science.1214824 PMC3914775

[b11] Sims P. C. . Electronic measurements of single-molecule catalysis by cAMP-dependent protein kinase a. J. Am. Chem. Soc. 135, 7861–7868 (2013). 23631749 10.1021/ja311604j PMC3738266

[b12] Davis J. J. Molecular bioelectronics. Philos. Trans. R. Soc. London, Ser. A 361, 2807–2825 (2003). 10.1098/rsta.2003.1270 14667299

[b13] Xu B., Xiao X. & Tao N. J. Measurements of single-molecule electromechanical properties. J. Am. Chem. Soc. 125, 16164–16165 (2003). 14692738 10.1021/ja038949j

[b14] Juhaniewicz J. & Sek S. Peptide molecular junctions: Distance dependent electron transmission through oligoprolines. Bioelectrochemistry 87, 21–27 (2012). 22197550 10.1016/j.bioelechem.2011.11.013

[b15] Sek S. Two metal−molecule binding modes for peptide molecular junctions. J. Phys. Chem. C 111, 12860–12865 (2007).

[b16] Juhaniewicz J. & Sek S. Peptide molecular junctions: Electron transmission through individual amino acid residues. J. Electroanal. Chem. 649, 83–88 (2010).

[b17] Nitzan A. Electron transmission through molecules and molecular interfaces. Annu. Rev. Phys. Chem. 52, 681–750 (2001). 11326078 10.1146/annurev.physchem.52.1.681

[b18] Moreno-García P. . Single-molecule conductance of functionalized oligoynes: Length dependence and junction evolution. J. Am. Chem. Soc. 135, 12228–12240 (2013). 23875671 10.1021/ja4015293

[b19] Simpkins S. M. E., Weller M. D. & Cox L. R. β-chlorovinylsilanes as masked alkynes in oligoyne assembly: Synthesis of the first aryl-end-capped dodecayne. Chem. Commun. 4035–4037 (2007). 10.1039/b707681a 17912407

[b20] Lopez-Perez D. E. . Intermolecular interactions in electron transfer through stretched helical peptides. Phys. Chem. Chem. Phys. 14, 10332–10344 (2012). 22735160 10.1039/c2cp40761b

[b21] Pawlowski J., Juhaniewicz J., Tymecka D. & Sek S. Electron transfer across α-helical peptide monolayers: Importance of interchain coupling. Langmuir 28, 17287–17294 (2012). 23181704 10.1021/la302716n

[b22] Li W. . Temperature and force dependence of nanoscale electron transport via the Cu protein azurin. ACS Nano 6, 10816–10824 (2012). 23136937 10.1021/nn3041705

[b23] Zahid F. . A self-consistent transport model for molecular conduction based on extended hückel theory with full three-dimensional electrostatics. J. Chem. Phys. 123, 064707 (2005). 10.1063/1.1961289 16122335

[b24] Taylor J., Brandbyge M. & Stokbro K. Conductance switching in a molecular device: The role of side groups and intermolecular interactions. Phys. Rev. B 68, 121101 (2003).

[b25] McConnell H. M. Intramolecular charge transfer in aromatic free radicals. J. Chem. Phys. 35, 508–515 (1961).

[b26] Marcus R. A. Electron transfer reactions in chemistry: Theory and experiment (nobel lecture). Angew. Chem. Int. Edit 32, 1111–1121 (1993).

[b27] Kai M., Takeda K., Morita T. & Kimura S. Distance dependence of long-range electron transfer through helical peptides. J. Pept. Sci. 14, 192–202 (2008). 18035857 10.1002/psc.974

[b28] Jortner J., Bixon M., Langenbacher T. & Michel-Beyerle M. E. Charge transfer and transport in DNA. Proc. Natl. Acad. Sci. USA 95, 12759–12765 (1998). 9788986 10.1073/pnas.95.22.12759 PMC23577

[b29] Arikuma Y., Nakayama H., Morita T. & Kimura S. Electron hopping over 100 along an α helix. Angew. Chem. Int. Edit 49, 1800–1804 (2010). 10.1002/anie.200905621 20155768

[b30] Onuchic J. N., Kobayashi C. & Baldridge K. K. Quantum tunneling in biological reactions: The interplay between theory and experiments. J. Braz. Chem. Soc. 19, 206–210 (2008).

[b31] Solomon G. C., Andrews D. Q., Van Duyne R. P. & Ratner M. A. When things are not as they seem: Quantum interference turns molecular electron transfer “rules” upside down. J. Am. Chem. Soc. 130, 7788–7789 (2008). 18517207 10.1021/ja801379b

[b32] Ricks A. B. . Controlling electron transfer in donor−bridge−acceptor molecules using cross-conjugated bridges. J. Am. Chem. Soc. 132, 15427–15434 (2010). 20942407 10.1021/ja107420a

[b33] de Andrade P. C. P. & Onuchic J. N. Generalized pathway model to compute and analyze tunneling matrix elements in proteins. J. Chem. Phys. 108, 4292–4298 (1998).

[b34] Miyashita O., Okamura M. Y. & Onuchic J. N. Interprotein electron transfer from cytochrome c2 to photosynthetic reaction center: Tunneling across an aqueous interface. Proc. Natl. Acad. Sci. USA 102, 3558–3563 (2005). 15738426 10.1073/pnas.0409600102 PMC553326

[b35] Petrov E. G., Shevchenko Y. V., Teslenko V. I. & May V. Nonadiabatic donor–acceptor electron transfer mediated by a molecular bridge: A unified theoretical description of the superexchange and hopping mechanism. J. Chem. Phys. 115, 7107–7122 (2001).

[b36] Isied S. S., Ogawa M. Y. & Wishart J. F. Peptide-mediated intramolecular electron transfer: Long-range distance dependence. Chem. Rev. 92, 381–394 (1992).

[b37] Mandal H. S. & Kraatz H.-B. Electron transfer mechanism in helical peptides. J. Phys. Chem. Lett. 3, 709–713 (2012). 26286277 10.1021/jz300008s

[b38] Mandal H. S. & Kraatz H.-B. Electron transfer across α-helical peptides: Potential influence of molecular dynamics. Chem. Phys. 326, 246–251 (2006).

[b39] Takeda K., Morita T. & Kimura S. Effects of monolayer structures on long-range electron transfer in helical peptide monolayer. J. Phys. Chem. B 112, 12840–12850 (2008). 18793017 10.1021/jp805711v

[b40] Zhang Y., Liu C., Balaeff A., Skourtis S. S. & Beratan D. N. Biological charge transfer via flickering resonance. Proc. Natl. Acad. Sci. USA 111, 10049–10054 (2014). 24965367 10.1073/pnas.1316519111 PMC4104919

[b41] Marcus R. A. On the theory of oxidation‐reduction reactions involving electron transfer. I. J. Chem. Phys. 24, 966–978 (1956).

[b42] Xiao X. Y., Xu B. Q. & Tao N. J. Conductance titration of single-peptide molecules. J. Am. Chem. Soc. 126, 5370–5371 (2004). 15113203 10.1021/ja049469a

[b43] Sek S. Review peptides and proteins wired into the electrical circuits: An SPM-based approach. Pept. Sci. 100, 71–81 (2013). 10.1002/bip.22148 23335169

[b44] Binnig G. & Rohrer H. Scanning tunneling microscopy. IBM. J. Res. Dev. 44, 279–293 (2000).

[b45] Giessibl F. J. Advances in atomic force microscopy. Rev. Mod. Phys. 75, 949–983 (2003).

[b46] Shah A. . Electron transfer in peptides. Chem. Soc. Rev. 44, 1015–1027 (2015). 25619931 10.1039/c4cs00297k

[b47] Polo F., Antonello S., Formaggio F., Toniolo C. & Maran F. Evidence against the hopping mechanism as an important electron transfer pathway for conformationally constrained oligopeptides. J. Am. Chem. Soc. 127, 492–493 (2005). 15643851 10.1021/ja043607e

[b48] Sek S., Palys B. & Bilewicz R. Contribution of intermolecular interactions to electron transfer through monolayers of alkanethiols containing amide groups. J. Phys. Chem. B 106, 5907–5914 (2002).

[b49] Malak R. A., Gao Z., Wishart J. F. & Isied S. S. Long-range electron transfer across peptide bridges: The transition from electron superexchange to hopping. J. Am. Chem. Soc. 126, 13888–13889 (2004). 15506726 10.1021/ja0401040

[b50] Sek S., Swiatek K. & Misicka A. Electrical behavior of molecular junctions incorporating α-helical peptide. J. Phys. Chem. B 109, 23121–23124 (2005). 16375270 10.1021/jp055709c

[b51] Cordes M. . Influence of amino acid side chains on long-distance electron transfer in peptides: Electron hopping via “stepping stones”. Angew. Chem. Int. Edit 47, 3461–3463 (2008). 10.1002/anie.200705588 18399515

[b52] Lin J., Balabin I. A. & Beratan D. N. The nature of aqueous tunneling pathways between electron-transfer proteins. Science 310, 1311–1313 (2005). 16311331 10.1126/science.1118316 PMC3613566

[b53] Migliore A., Corni S., Di Felice R. & Molinari E. Water effects on electron transfer in azurin dimers. J. Phys. Chem. B 110, 23796–23800 (2006). 17125342 10.1021/jp064690q

[b54] Di Valentin M. . Evidence for water-mediated triplet–triplet energy transfer in the photoprotective site of the peridinin–chlorophyll a–protein. BBA. Bioenergetics 1837, 85–97 (2014). 23871938 10.1016/j.bbabio.2013.07.005

[b55] Chakrabarti S., Parker M. F. L., Morgan C. W., Schafmeister C. E. & Waldeck D. H. Experimental evidence for water mediated electron transfer through bis-amino acid donor−bridge−acceptor oligomers. J. Am. Chem. Soc. 131, 2044–2045 (2009). 19173584 10.1021/ja8079324 PMC3210553

[b56] Migliore A., Corni S., Di Felice R. & Molinari E. Water-mediated electron transfer between protein redox centers. J. Phys. Chem. B 111, 3774–3781 (2007). 17388538 10.1021/jp068773i

[b57] Langen R. . Electron tunneling in proteins: Coupling through a beta strand. Science 268, 1733–1735 (1995). 7792598 10.1126/science.7792598

[b58] Tao N. J. Electron transport in molecular junctions. Nat. Nano 1, 173–181 (2006). 10.1038/nnano.2006.130 18654182

[b59] Weinkauf R., Schanen P., Yang D., Soukara S. & Schlag E. W. Elementary processes in peptides: Electron mobility and dissociation in peptide cations in the gas phase. J. Phys. Chem. 99, 11255–11265 (1995).

[b60] Baranov L. Y. & Schlag E. W. New mechanism for facile charge transport in polypeptides. Z. Naturforsch. A 54, 387–396 (1999).

[b61] Sheu S.-Y., Schlag E. W. & Yang D.-Y. A model for ultra-fast charge transport in membrane proteins. Phys. Chem. Chem. Phys. 17, 23088–23094 (2015). 26274051 10.1039/c5cp01442e

[b62] Nick Pace C. & Martin Scholtz J. A helix propensity scale based on experimental studies of peptides and proteins. Biophys. J. 75, 422–427 (1998). 9649402 10.1016/s0006-3495(98)77529-0 PMC1299714

[b63] Chen Z.-w., Barber M. J., McIntire W. S. & Mathews F. S. Crystallographic study of azurin from pseudomonas putida. Acta Crystallogr. Sect. D 54, 253–268 (1998). 9761890 10.1107/s0907444997011505

[b64] de Rege P. J., Williams S. A. & Therien M. J. Direct evaluation of electronic coupling mediated by hydrogen bonds: Implications for biological electron transfer. Science 269, 1409–1413 (1995). 7660123 10.1126/science.7660123

[b65] Warren J. J., Herrera N., Hill M. G., Winkler J. R. & Gray H. B. Electron flow through nitrotyrosinate in pseudomonas aeruginosa azurin. J. Am. Chem. Soc. 135, 11151–11158 (2013). 23859602 10.1021/ja403734n PMC3839300

[b66] Dempsey J. L., Winkler J. R. & Gray H. B. Proton-coupled electron flow in protein redox machines. Chem. Rev. 110, 7024–7039 (2010). 21082865 10.1021/cr100182b PMC3005815

[b67] Gray H. B. & Winkler J. R. Electron flow through metalloproteins. BBA. Bioenergetics 1797, 1563–1572 (2010). 20460102 10.1016/j.bbabio.2010.05.001

[b68] Collman J. P. . A cytochrome c oxidase model catalyzes oxygen to water reduction under rate-limiting electron flux. Science 315, 1565–1568 (2007). 17363671 10.1126/science.1135844 PMC3064436

[b69] Matthews C. R., Erickson P. M., Van Vliet D. L. & Petersheim M. Synthesis of pentaammineruthenium-histidine complexes in ribonuclease a. J. Am. Chem. Soc. 100, 2260–2262 (1978).

[b70] Bjerrum M. . Electron transfer in ruthenium-modified proteins. J. Bioenerg. Biomembr. 27, 295–302 (1995). 8847343 10.1007/BF02110099

[b71] Frisch M. J. . Gaussian 09 Revision D.01 (Gaussian, Inc, Wallingford, CT, USA, 2009).

[b72] Schlag E. W., Sheu S. Y., Yang D. Y., Selzle H. L. & Lin S. H. Distal charge transport in peptides. Angew. Chem. Int. Edit 46, 3196–3210 (2007). 10.1002/anie.200601623 17372995

[b73] Nishino T., Hayashi N. & Bui P. T. Direct measurement of electron transfer through a hydrogen bond between single molecules. J. Am. Chem. Soc. 135, 4592–4595 (2013). 23488642 10.1021/ja311463b

[b74] Vyalikh D. V. . Charge transport in proteins probed by resonant photoemission. Phys. Rev. Lett. 102, 098101 (2009). 19392567 10.1103/PhysRevLett.102.098101

[b75] Kettner M. . Spin filtering in electron transport through chiral oligopeptides. J. Phys. Chem. C 119, 14542–14547 (2015).

[b76] Jacob D. & Palacios J. J. Orbital eigenchannel analysis for ab initio quantum transport calculations. Phys. Rev. B 73, 075429 (2006).

[b77] Mahan G. D. Many-Particle Physics, Third ed. (Plenum Publishers, 2000).

[b78] Johnson M. & Silsbee R. H. Thermodynamic analysis of interfacial transport and of the thermomagnetoelectric system. Phys. Rev. B 35, 4959–4972 (1987). 10.1103/physrevb.35.4959 9940677

[b79] Bogani L. & Wernsdorfer W. Molecular spintronics using single-molecule magnets. Nat. Mater. 7, 179–186 (2008). 18297126 10.1038/nmat2133

[b80] An Y. & Yang Z. Spin-filtering and switching effects of a single-molecule magnet mn(dmit)2. J. Appl. Phys. 111, 043713 (2012).

[b81] Huang J., Wang W., Yang S., Li Q. & Yang J. Efficient spin filter based on FeN_4_ complexes between carbon nanotube electrodes. Nanotechnology 23, 255202 (2012). 22652524 10.1088/0957-4484/23/25/255202

