# Supplementary material for: Mechanically Controlled Electron Transfer in a Single-Polypeptide Transistor
Source: Sci Rep. 2017 Jan 4;7:39792. doi: 10.1038/srep39792 (PMC5209712; doi:10.1038/srep39792)
Supplement: Supplementary Data [file srep39792-s1.pdf]

Supplementary data

Mechanically Controlled Electron Transfer in a Single-Polypeptide Transistor

Sheh-Yi Sheu[1,†,*] and Dah-Yen Yang[2,†,*]

[1] Department of Life Sciences, Institute of Genome Sciences and Institute of Biomedical Informatics, National Yang-Ming University, Taipei 112, Taiwan

[2] Institute of Atomic and Molecular Sciences, Academia Sinica, Taipei 106, Taiwan

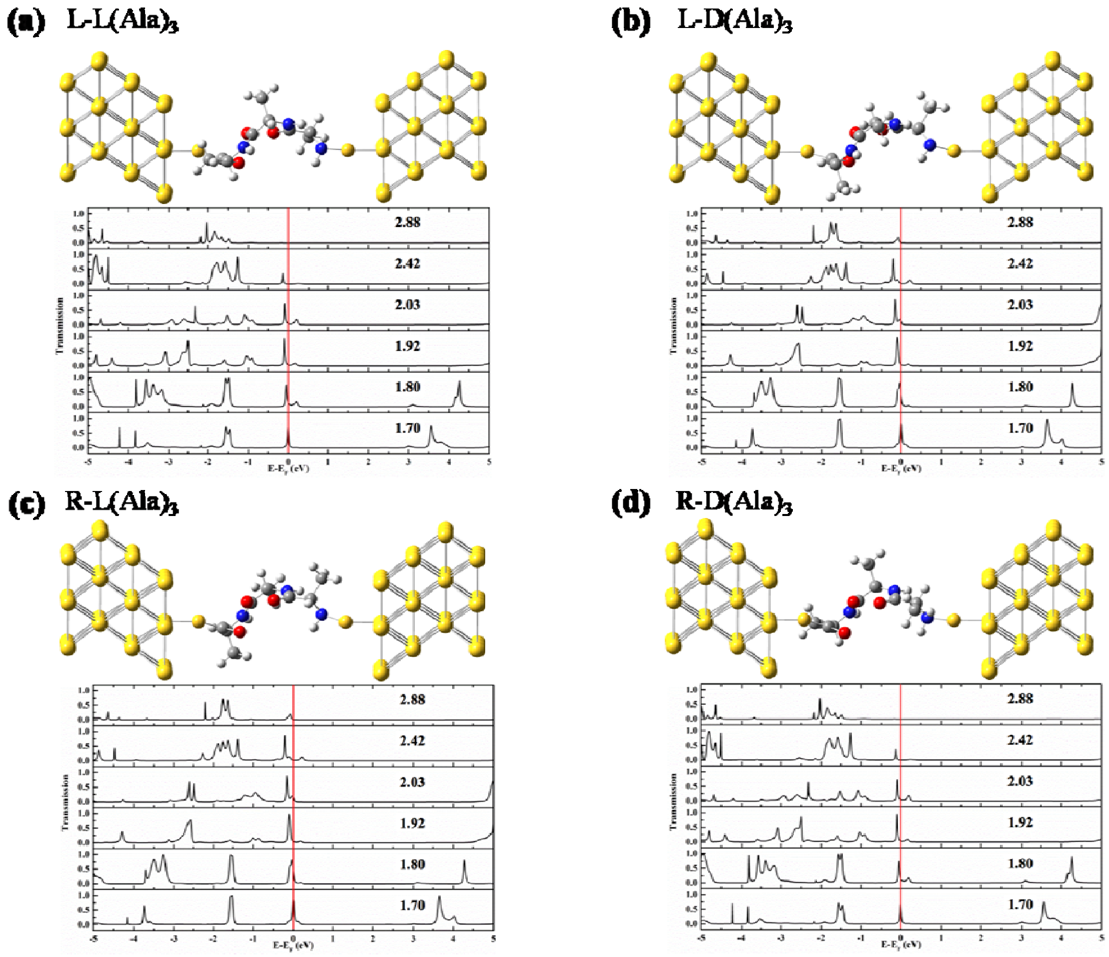
(a) L-L(Ala)3
(b) L-D(Ala)3
(c) R-L(Ala)3
(d) R-D(Ala)3
2.88
2.42
2.03
1.92
1.80
1.70
Transmission
E-EF (eV)

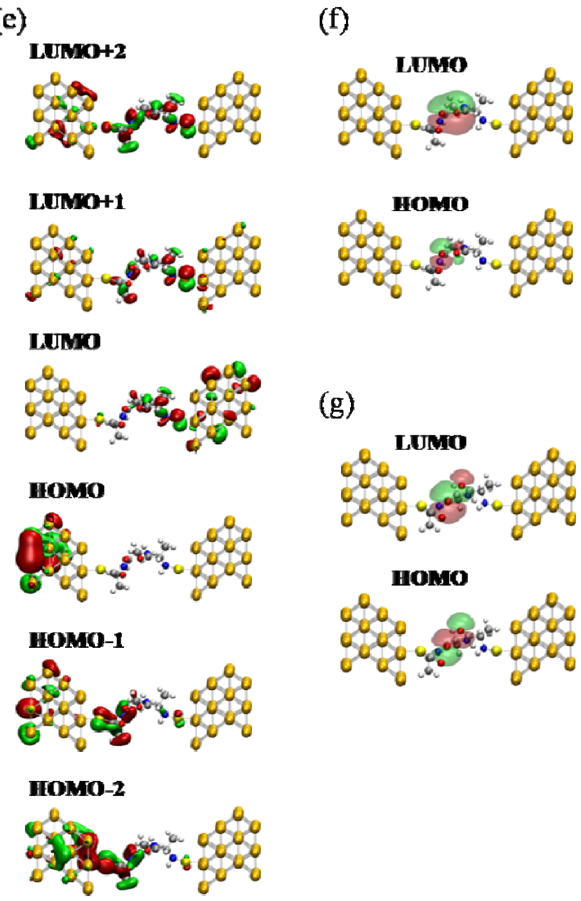

Figure S1. Molecular junctions and TS of the four enantiomers (Ala)$_3$. (a) L-L(Ala)$_3$, (b) L-D(Ala)$_3$, (c) R-L(Ala)$_3$ and (d) R-D(Ala)$_3$. The molecular junction (Ala)$_3$ (O: red, N: blue, C: gray and H: light gray) was wired to the Au electrodes (golden yellow) through the S atom (brown). The source, scatter and drain components are the left electrode, the polypeptide and the right electrode, respectively. TS versus d_{O-O}: 1.70, 1.80, 1.92, 2.03, 2.42 and 2.88 Å. (e) Eigenchannel in the molecular orbital representation. The orbital is represented as positive (green) and negative (red) intensities. The isovalue is 0.005. Eigenchannel in the atomic orbital representation at (f) d_{O-O} = 1.92 Å and (g) d_{O-O} = 2.42 Å. The isovalue is 0.001.

(a) α helix

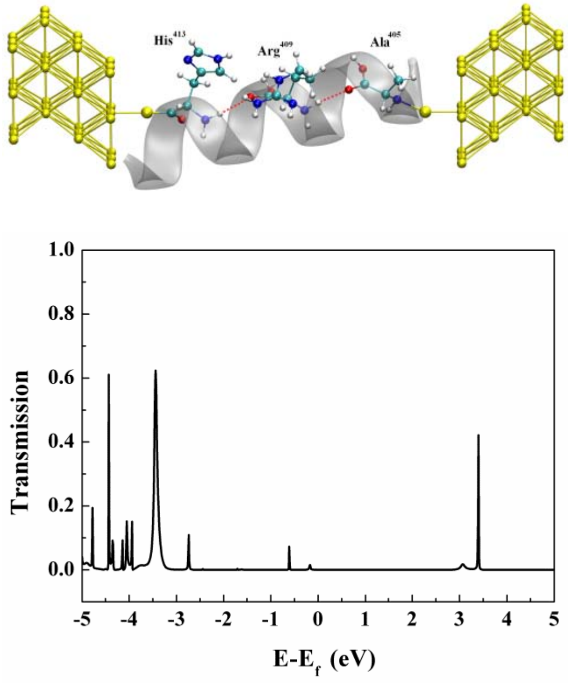

(f) Parallel β sheet

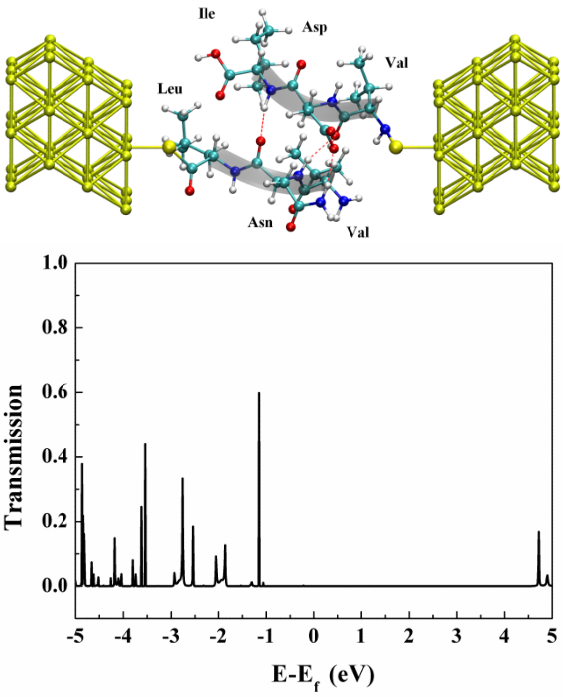

(b) α helix + 1 Cl^{-1}

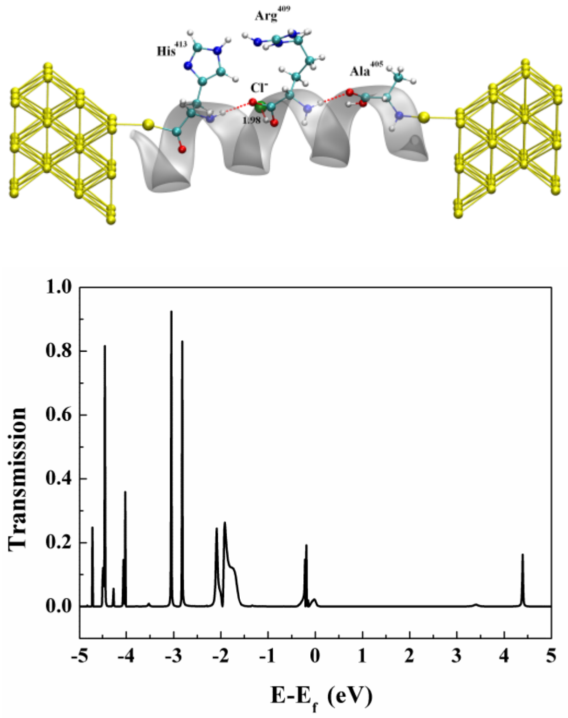

(g) Parallel β sheet + 3 Cl^{-1}

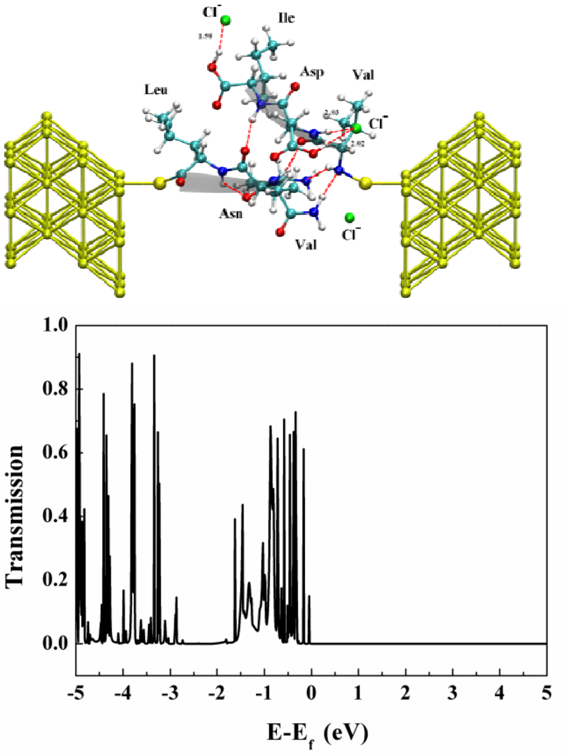

(c) α helix + 1 Cl^{-1} + 5 H_2O

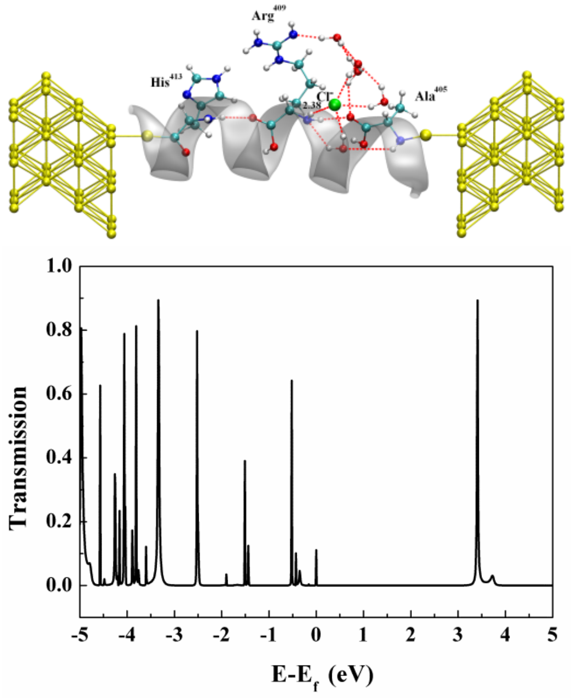

(h) Parallel β sheet + 15 H_2O

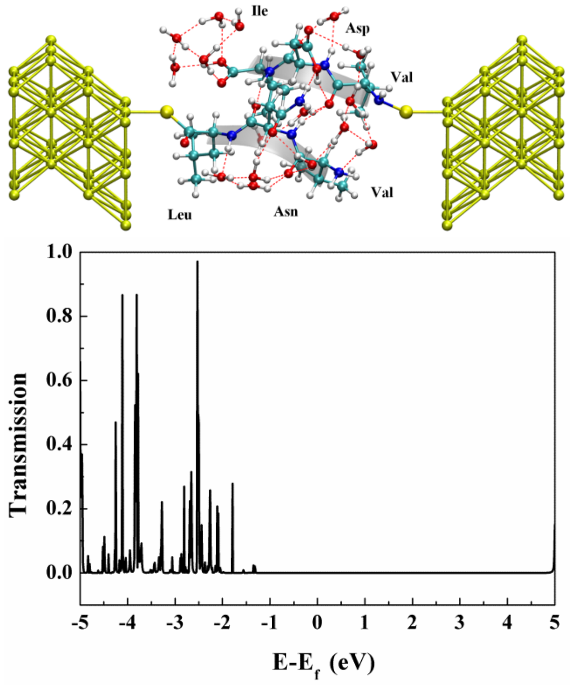

(d) α helix + 3 Cl^{-}

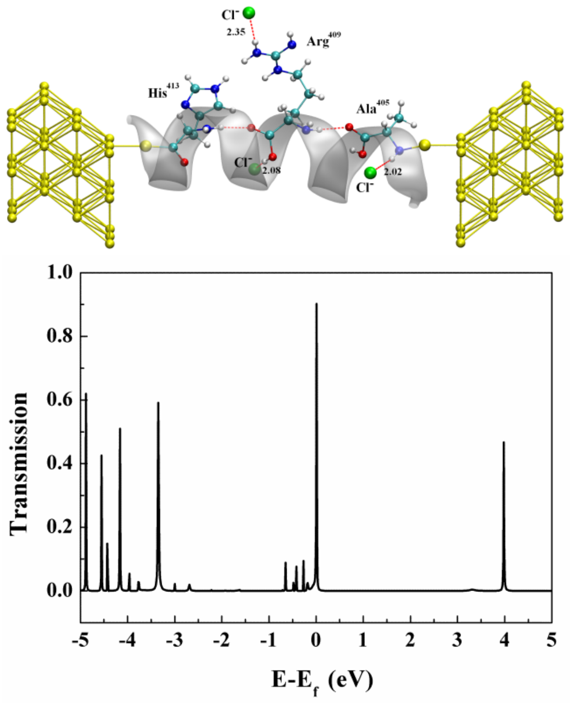

(i) Anti-parallel β sheet

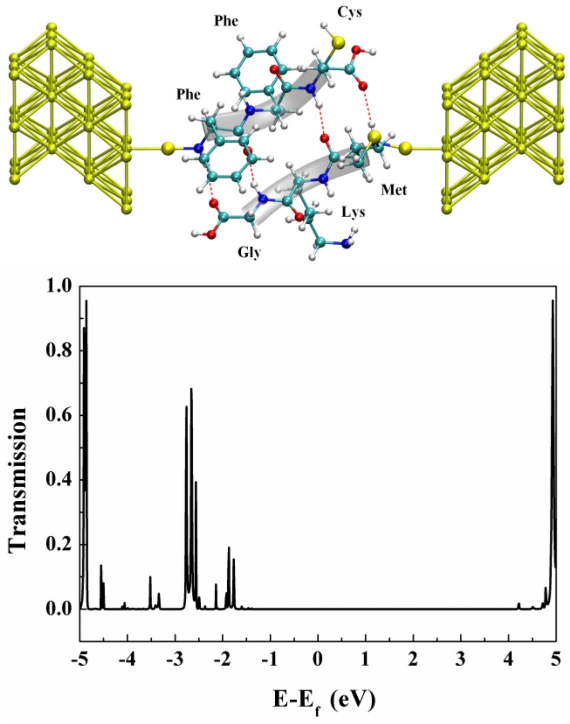

(e) α helix + 13 H_2O

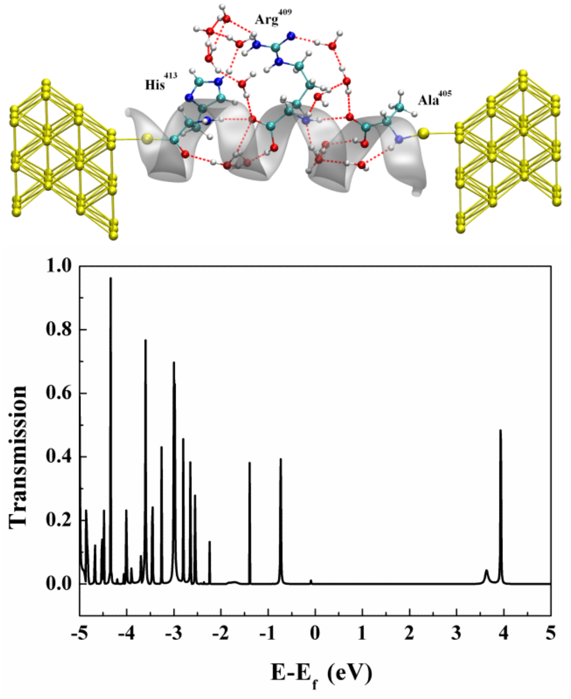

(j) Anti-parallel β sheet + 1 Cl^{-1} + 3 H_2O

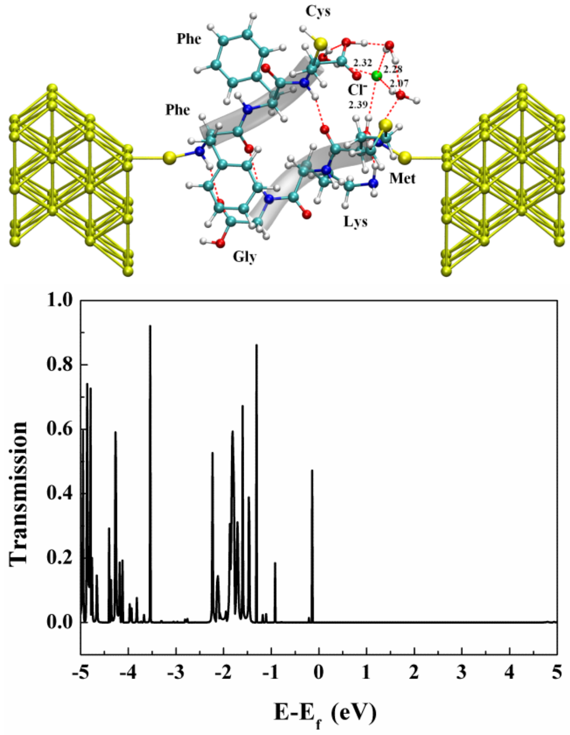

(k) Anti-parallel β sheet +H_2O

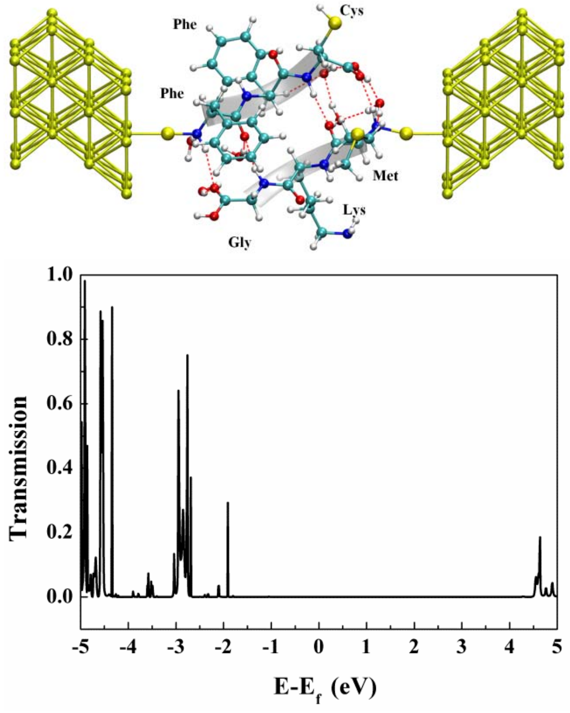

(l)

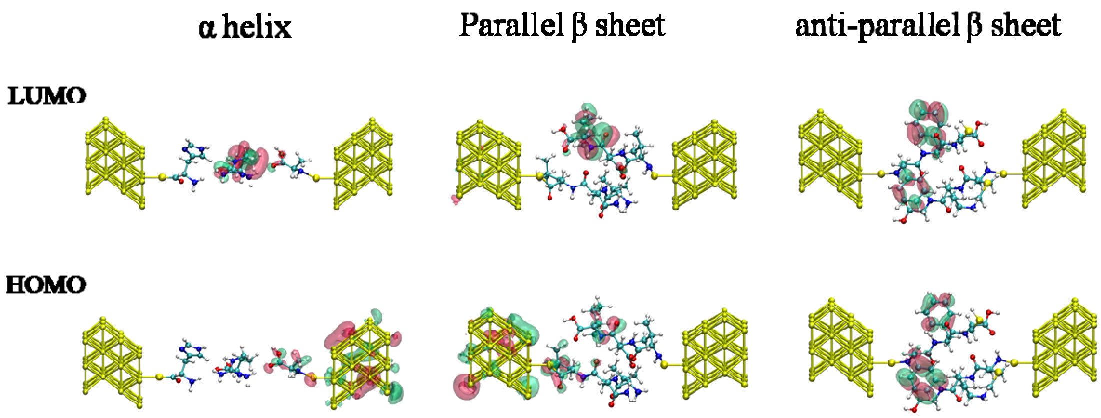

Figure S2. TS of α helix and parallel and anti-parallel β sheets. (a-k) TS with respect to the molecular junction system in the presence of Cl^{-1} ions (in green) and water molecules. The ribbon representation in the α helix is provided to facilitate visualization. A single molecular junction: peptide (O: red, N: blue, C: gray and H: light gray) was wired to the Au electrodes (yellow) through the interfacial S atom (brown). A water molecule is shown in a ball-and-stick representation and color-coded by atom type**. (l)** Molecular orbital structures of the α helix and parallel and anti-parallel β sheets.

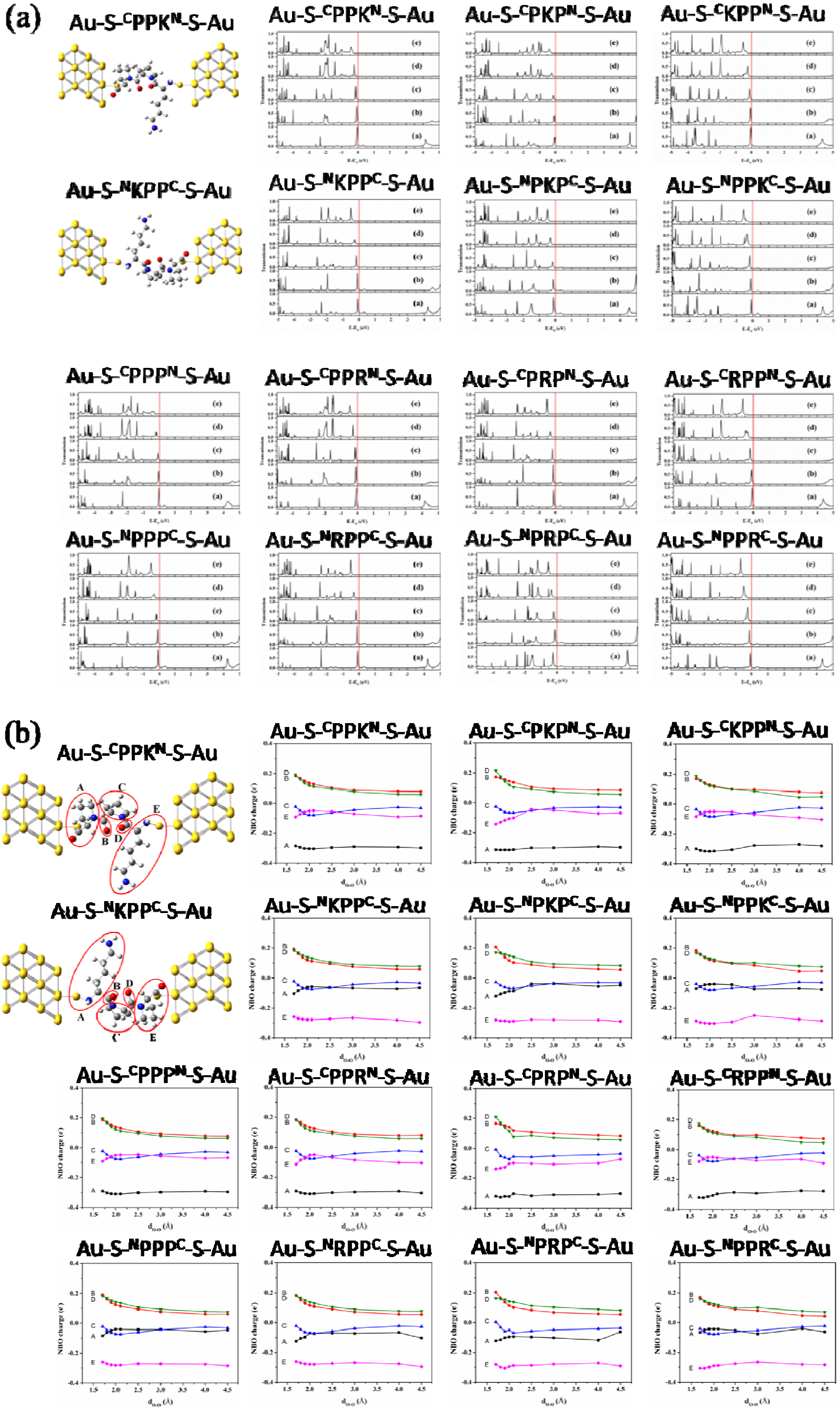
(a)
Au-S-^{C}PPK^{N}-S-Au
Au-S-^{C}PKP^{N}-S-Au
Au-S-^{C}KPP^{N}-S-Au
Au-S-^{N}KPP^{C}-S-Au
Au-S-^{N}PKP^{C}-S-Au
Au-S-^{N}PPK^{C}-S-Au
Au-S-^{C}PPP^{N}-S-Au
Au-S-^{C}PPR^{N}-S-Au
Au-S-^{C}PRP^{N}-S-Au
Au-S-^{C}RPP^{N}-S-Au
Au-S-^{N}PPP^{C}-S-Au
Au-S-^{N}RPP^{C}-S-Au
Au-S-^{N}PRP^{C}-S-Au
Au-S-^{N}PPR^{C}-S-Au
(b)
Au-S-^{C}PPK^{N}-S-Au
Au-S-^{C}PKP^{N}-S-Au
Au-S-^{C}KPP^{N}-S-Au
Au-S-^{N}KPP^{C}-S-Au
Au-S-^{N}PKP^{C}-S-Au
Au-S-^{N}PPK^{C}-S-Au
Au-S-^{C}PPP^{N}-S-Au
Au-S-^{C}PPR^{N}-S-Au
Au-S-^{C}PRP^{N}-S-Au
Au-S-^{C}RPP^{N}-S-Au
Au-S-^{N}PPP^{C}-S-Au
Au-S-^{N}RPP^{C}-S-Au
Au-S-^{N}PRP^{C}-S-Au
Au-S-^{N}PPR^{C}-S-Au

Figure S3. TS and NBO charge distribution of the various PPP, KPP, and RPP peptides. **(a)** The molecular junction structures and TS versus d_{O-O} (a) 1.70, (b) 1.80, (c) 1.90, (d) 2.00 and (e) 2.10 Å. **(b)** NBO charge distribution versus d_{O-O} at the five regions (A, B, C, D and E) of the various PPP, KPP, and RPP peptides. The notations are identical to those in the legend of Fig. S1. For example, the molecular junction system is denoted by Au-S-^{C}PPK^{N}-S-Au and Au-S-^{N}PPK^{C}-S-Au, where ^{C}PPK^{N} and ^{N}PPK^{C} indicate the N→C and C→N direction, respectively.

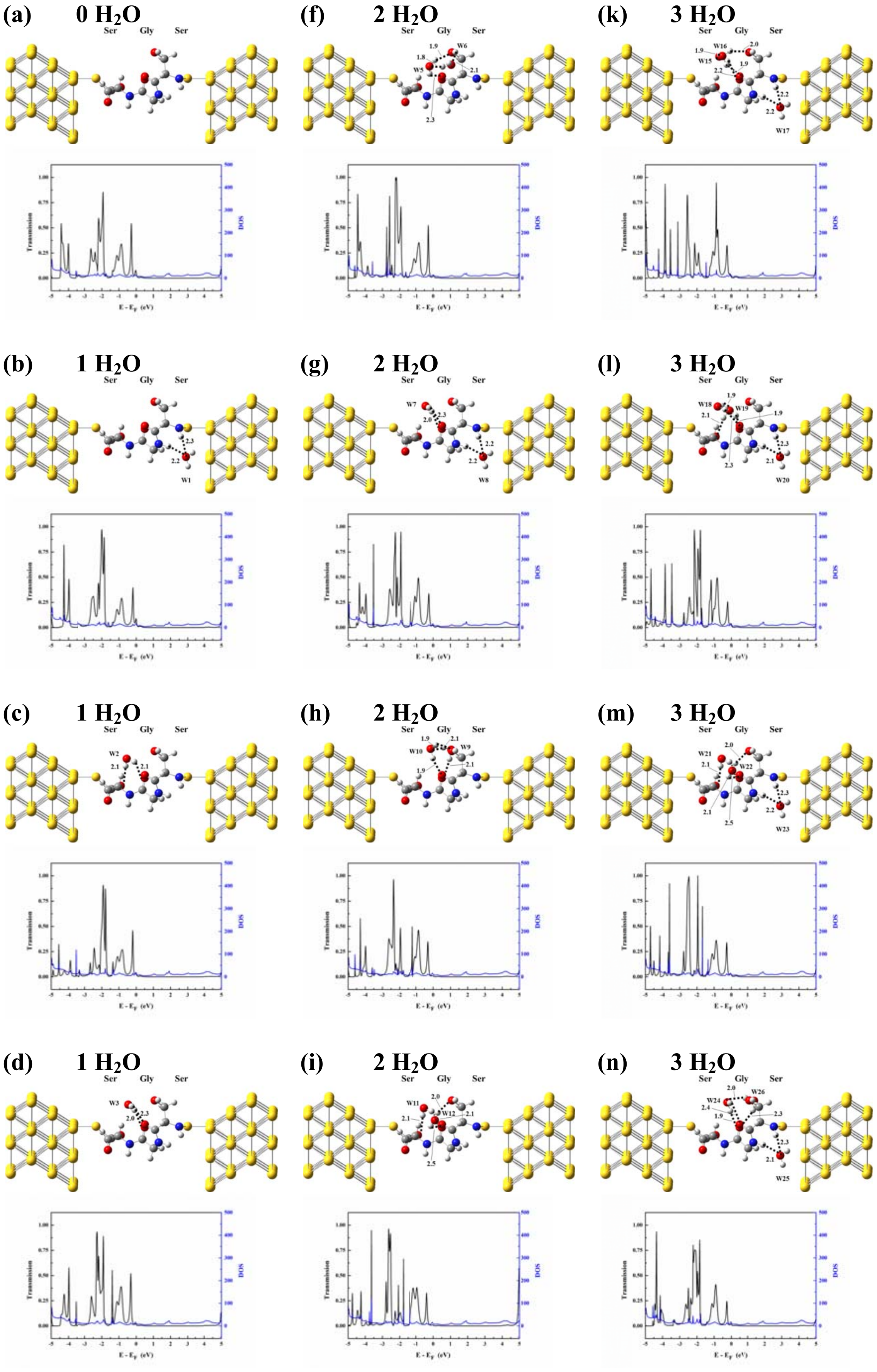
(a) 0 H_2O
Ser Gly Ser
(f) 2 H_2O
Ser Gly Ser
(k) 3 H_2O
Ser Gly Ser
(b) 1 H_2O
Ser Gly Ser
W1
(g) 2 H_2O
Ser Gly Ser
W7
W8
(l) 3 H_2O
Ser Gly Ser
W18
W19
W20
(c) 1 H_2O
Ser Gly Ser
W2
(h) 2 H_2O
Ser Gly Ser
W10
W9
(m) 3 H_2O
Ser Gly Ser
W21
W22
W23
(d) 1 H_2O
Ser Gly Ser
W3
(i) 2 H_2O
Ser Gly Ser
W11
W12
(n) 3 H_2O
Ser Gly Ser
W24
W26
W25
Transmission
DOS
E - E$_F$ (eV)

(e) 1 H_2O

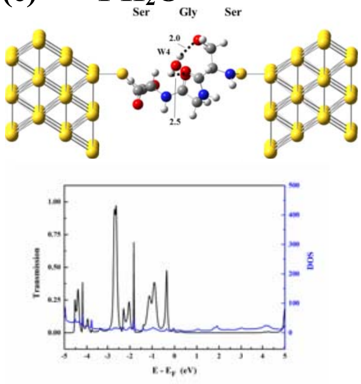

(j) 2 H_2O

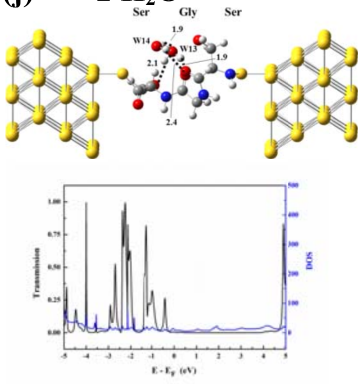

(o) 3 H_2O

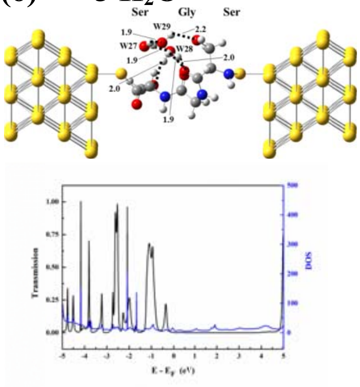

(p) 3 H_2O

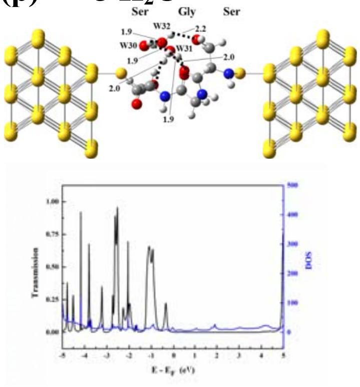

(q) 3 H_2O

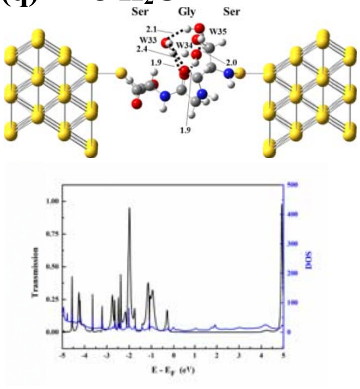

Figure S4. The molecular junction systems and TS. **(a-q)** The peptide SGS in the presence of water molecules, nH_2O (n=0, 1, 2 and 3). The notations are identical to those in the legend of **Figure S1**.

Table S1. **Atomic orbital coefficients of the HOMO and LUMO.** $\phi_{a,b}$ denotes the atomic orbital, where *a* is the atom type and number and *b* indicates the Gaussian basis set.

(a) $d_{O-O} = 1.92$ Å

$\Phi_{MO}^{LUMO+3} = -0.018\times\phi_{O_{48},2p_x} + 0.019\times\phi_{O_{48},2p_y} - 0.014\times\phi_{O_{48},3s} - 0.011\times\phi_{O_{48},3p_x}$
$\Phi_{MO}^{LUMO+2} = +0.014\times\phi_{O_{41},2p_x} + 0.014\times\phi_{O_{41},2p_z} + 0.012\times\phi_{O_{41},3p_z} - 0.02\times\phi_{O_{48},2p_x}$<br>$+ 0.013\times\phi_{O_{48},2p_y} - 0.029\times\phi_{O_{48},2p_z} - 0.011\times\phi_{O_{48},3s} - 0.011\times\phi_{O_{48},3p_x}$<br>$- 0.014\times\phi_{O_{48},3p_z}$
$\Phi_{MO}^{LUMO+1} = 0.012\times\phi_{O_{41},2p_x} + 0.018\times\phi_{O_{48},2p_x} + 0.015\times\phi_{O_{48},2p_z} + 0.010\times\phi_{O_{48},3p_x}$
$\Phi_{MO}^{LUMO} = +0.018\times\phi_{O_{41},2p_x} + 0.010\times\phi_{O_{41},3p_x}$
$\Phi_{MO}^{HOMO} = \text{coefficient} < 0.01$
$\Phi_{MO}^{HOMO-1} = -0.01\times\phi_{O_{41},2p_z} + 0.031\times\phi_{O_{48},2p_x} - 0.019\times\phi_{O_{48},2p_y} + 0.021\times\phi_{O_{48},2p_z}$<br>$+ 0.016\times\phi_{O_{48},3p_x}$
$\Phi_{MO}^{HOMO-2} = -0.028\times\phi_{O_{48},2p_x} + 0.016\times\phi_{O_{48},2p_y} - 0.023\times\phi_{O_{48},2p_z} - 0.014\times\phi_{O_{48},3p_x}$<br>$- 0.011\times\phi_{O_{48},3p_z}$
$\Phi_{MO}^{HOMO-3} = +0.017\times\phi_{O_{41},2s} + 0.017\times\phi_{O_{41},2p_x} + 0.016\times\phi_{O_{41},2p_y} - 0.019\times\phi_{O_{41},2p_z}$<br>$+ 0.031\times\phi_{O_{41},3s} - 0.01\times\phi_{O_{41},3p_z}$

(b) $d_{O-O} = 2.42$ Å

$\Phi_{MO}^{LUMO+3} = +0.025\times\phi_{O_{48},2p_x} + 0.014\times\phi_{O_{48},2p_y} + 0.025\times\phi_{O_{48},2p_z} + 0.015\times\phi_{O_{48},3p_x}$<br>$+ 0.010\times\phi_{O_{48},3p_y} + 0.011\times\phi_{O_{48},3p_z}$
$\Phi_{MO}^{LUMO+2} = +0.011\times\phi_{O_{41},2s} - 0.034\times\phi_{O_{41},2p_x} + 0.011\times\phi_{O_{41},2p_y} - 0.027\times\phi_{O_{41},3p_x}$
$\Phi_{MO}^{LUMO+1} = +0.021\times\phi_{O_{48},2p_x} + 0.011\times\phi_{O_{48},2p_z} + 0.012\times\phi_{O_{48},3p_x}$
$\Phi_{MO}^{LUMO} = +0.010\times\phi_{O_{48},2p_x}$
$\Phi_{MO}^{HOMO} = -0.013\times\phi_{O_{41},2p_z}$
$\Phi_{MO}^{HOMO-1} = +0.035\times\phi_{O_{48},2p_x} + 0.021\times\phi_{O_{48},2p_z} + 0.019\times\phi_{O_{48},3p_x} + 0.010\times\phi_{O_{48},3p_z}$
$\Phi_{MO}^{HOMO-2} = +0.016\times\phi_{O_{41},2p_x} + 0.011\times\phi_{O_{41},3p_x} + 0.011\times\phi_{O_{48},2s} - 0.025\times\phi_{O_{48},2p_y}$<br>$- 0.02\times\phi_{O_{48},2p_z} + 0.014\times\phi_{O_{48},3s} - 0.015\times\phi_{O_{48},3p_y}$
$\Phi_{MO}^{HOMO-3} = +0.041\times\phi_{O_{41},2p_x} - 0.016\times\phi_{O_{41},2p_y} - 0.02\times\phi_{O_{41},2p_z} + 0.035\times\phi_{O_{41},3s}$<br>$+ 0.030\times\phi_{O_{41},3p_x} - 0.01\times\phi_{O_{41},3p_z}$

Table S2. **NBO charge distribution on water molecules**

type		Water bridge		
		NHO	COHO	OHO
structure		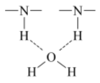	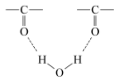	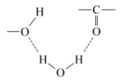
system		Natural Charge (e^-) of H_2O		
SGS + 0 H_2O	A			
SGS + 1 H_2O	B	0.06218		
	C			-0.01480
	D		0.00720	
	E			-0.01789
SGS + 2 H_2O	F		0.01434	-0.02196
	G	0.06425	0.01186	
	H		0.01714	-0.01658
	I			-0.01842<br>-0.01144
	J		0.00420	-0.00499
SGS + 3 H_2O	K	0.05982	0.00606	-0.01720
	L	0.06358	0.00797	-0.00926
	M	0.06275		-0.01030<br>-0.01691
	N	0.06067	0.02459	-0.00473
	O		0.00545	-0.00011<br>-0.01381
	P		0.00440	-0.00058<br>-0.01325
	Q		0.01420<br>0.03665	-0.02705
